# Component-Level Cost and Variability Drivers of Intradetrusor OnabotulinumtoxinA Injections Across Office and Operating Room Settings

**DOI:** 10.1097/UPJ.0000000000001067

**Published:** 2026-09-03

**Authors:** Saawan D. Patel, Evan Rosario, Robert Stetson, Ariana L. Smith

**Affiliations:** 1 Perelman School of Medicine, University of Pennsylvania, Philadelphia, Pennsylvania; 2 Department of Strategic Decision Support, University of Pennsylvania, Philadelphia, Pennsylvania; 3 Division of Urology, Perelman School of Medicine, University of Pennsylvania, Philadelphia, Pennsylvania

**Keywords:** urinary bladder, overactive, botulinumtoxins, type A, costs and cost analysis, ambulatory surgical procedures, operating rooms

## Abstract

**Introduction:**

The purpose of this study was to identify component-level drivers of cost variability for intradetrusor onabotulinumtoxinA (BTX-A) injections for overactive bladder across office-based clinic, tertiary operating room (OR), and hospital outpatient department (HOPD) settings.

**Methods:**

We performed a retrospective cost analysis of BTX-A injection encounters from January 2020 to June 2025 using institutional activity-based cost accounting data. Costs were grouped into procedural components and categorized as direct or indirect. Mean costs were compared using Welch *t* tests with Bonferroni correction. Utilization-adjusted variance decomposition identified component-level drivers of cost variability.

**Results:**

The cohort included 322 tertiary OR, 125 HOPD, and 1492 clinic encounters. Mean total costs were $4633.87 in the tertiary OR, $5063.32 in the HOPD, and $1899.01 in clinic. Total cost variability differed by setting. In the tertiary OR, variability was driven by supplies (29.5%), OR time (25.7%), and postanesthesia recovery (25.5%), while direct cost variability was dominated by supplies (45.1%) and indirect variability by OR time (56.0%). In the HOPD, total cost variability was driven by OR time (41.2%), anesthesia (29.2%), and recovery (16.1%), with similar drivers of indirect variability. In the clinic, total and direct cost variability were driven primarily by cystoscopy (63.9% and 68.7%) and BTX-A (36.1% and 31.3%), while indirect variability was driven by BTX-A (65.4%).

**Conclusions:**

Cost variability differs substantially across procedural settings. Cost-containment efforts should target setting-specific variance drivers rather than mean costs or site of service alone.

The US spends substantially more on health care per capita than other high-income nations despite similar utilization rates, largely driven by higher prices for labor, supplies, pharmaceuticals, and administration.^1,2^ In 2024, the Organisation for Economic Co-operation and Development estimated that the US spent $14,885 per capita compared with the average of $5967.^3^ Surgical care accounts for nearly half of all Medicare payments, with urology leading with the fifth highest procedural volume.^4,5^ One national spending study estimated that of the $3.1 trillion spent on health care in 2016, $86 billion was attributed to urinary diseases.^6^ Despite increasing input costs, inflation-adjusted Medicare reimbursement for urologic procedures has declined by 47.44% from 2015 to 2024.^7^

**UPJ Insight**

**Study Need and Importance**
Intradetrusor onabotulinumtoxinA (BTX-A) is a guideline-supported, repeat procedural therapy for refractory overactive bladder. Although BTX-A injections may be performed in office-based clinics, tertiary operating rooms (ORs), or hospital outpatient departments (HOPDs), these sites differ substantially in facility resources, anesthesia, recovery, staffing, and overhead. Existing economic analyses have largely focused on treatment-level cost-effectiveness rather than identifying which procedural components drive cost variability. We evaluated component-level and variance drivers of costs for BTX-A injections from the health care provider perspective.
**What We Found**
Among 1939 encounters, mean total costs were $4633.87 in the tertiary OR, $5063.32 in the HOPD, and $1899.01 in the clinic. More importantly, the components driving cost variability differed by setting, as demonstrated in the Figure. In the tertiary OR, total cost variability was largely driven by supplies, OR time, and postanesthesia recovery. In the HOPD, variability was driven by OR time, anesthesia, and postanesthesia recovery. In the clinic, variability was concentrated more in cystoscopy-associated costs than in BTX-A.
**Limitations**
This single-institution analysis may not generalize to health systems with different purchasing contracts, staffing models, facility designations, or overhead allocation methods. Patient-level reasons for site selection were not reliably captured, so some variability may reflect appropriate use of higher-resource settings rather than inefficiency. Because our institution does not have a freestanding ambulatory surgery center, such encounters were not captured in this analysis.
**Interpretation for Patient Care**
Office-based BTX-A injection was substantially less costly, but these findings do not advocate for a uniform shift toward lower-acuity settings for all patients. Patient-specific factors such as need for anesthesia, procedural tolerance, and anatomic complexity should drive appropriate site utilization. Cost-containment efforts should therefore focus on modifiable drivers of variability rather than restricting access to higher-resource care when clinically indicated.

**Figure.**
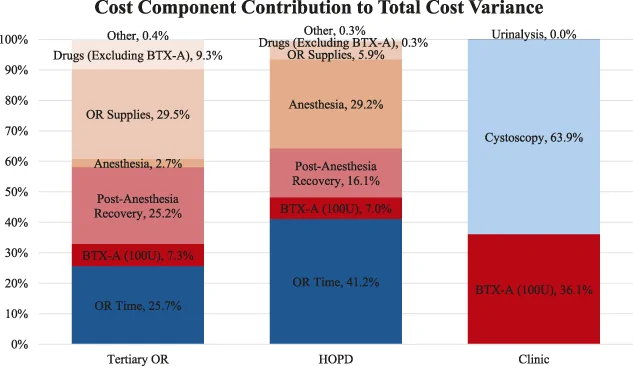
Relative contribution of cost components to total cost variance by setting, accounting for differences in utilization rates. The “other” category includes preoperative, laboratory, and electrocardiogram costs. BTX-A indicates onabotulinumtoxinA; HOPD, hospital outpatient department; OR, operating room.

To curb specialty care expenditures, there has been a cumulative movement in alternative payment strategies. While bundled payments have had limited penetration into urologic care, given little evidence that these models affect decision-making and cost cutting,^8,9^ novel efforts for urology-specific bundles may serve as potential solutions in the era of reimbursement reform.^10,11^ In light of these upcoming models and decreasing reimbursement under historical models, providers face increasing cost-containment pressure despite rising administrative, supply, and drug costs.

Overactive bladder (OAB) provides a relevant clinical context in which to examine procedural cost containment in urology. OAB affects over 33 million US adults, with prevalence exceeding 30% in those older than 65 years, and imposes an estimated $656 to $860 per patient on OAB-specific costs alone.^12^ Intradetrusor onabotulinumtoxinA (BTX-A) is a guideline-supported advanced therapy for refractory OAB and is frequently performed as a repeat intervention.^13^ BTX-A injections may be performed in either an office-based clinic or operating room (OR), using the same therapeutic agent but with different requirements for facility resources, anesthesia, recovery, staffing, and overhead. As a result, intradetrusor BTX-A offers a useful model for distinguishing drug-related costs from site-of-service and workflow-related cost drivers across settings.

Existing economic analyses in OAB have primarily focused on its superior cost-effectiveness profile across OAB therapies.^14-17^ However, there are no data identifying procedure-specific cost components and sources of variability for intradetrusor BTX-A. Mean costs identify which setting or component is more expensive, but they do not explain which components may be most amenable to intervention. Understanding these drivers is essential for targeting cost-reduction strategies without restricting access for patients who require more resource-intensive settings. We therefore performed a retrospective cost analysis of intradetrusor BTX-A injections performed in the office-based clinic, tertiary OR, and hospital outpatient department (HOPD) settings. We compared total, direct, and indirect costs by setting and characterized component-level contributions to identify high-cost and high-variability targets for cost-reduction strategies.

## Methods

### Patient Population

We performed a retrospective review of all patients undergoing intradetrusor BTX-A injection billed using current procedural terminology (CPT) 52287 (Cystourethroscopy, with injection[s] for chemodenervation of the bladder) at a single academic institution from January 2020 to June 2025. Our query pulled cases from office-based clinic, tertiary OR, and HOPD settings. Patients undergoing additional procedures within the same encounter were excluded. Institutional Review Board approval was obtained for this study.

### Cost Data and Analyses

Costs were analyzed from the perspective of a health care provider. Clinic and perioperative cost data were obtained from the University of Pennsylvania Health System Finance Department cost accounting system (STRATA, Roper Technologies), which delineates itemized costs by total, direct, and indirect costs per encounter. Clinic encounters were captured at the CPT/encounter level, whereas OR-based encounters included specific item-level cost accounting for facility, perioperative, and supply-related services. Site of service was clinically determined based on patient sedation requirements, limited mobility or positioning tolerance, anatomic considerations, significant procedural discomfort, or prior intolerance of office-based procedures. Each encounter was treated as a separate event because patients can undergo multiple BTX-A events. Only costs related to BTX-A injection, including those related to preprocedural testing, were included. Using UB-04 (Uniform Bill of 2004) codes, OR costs were categorized into expenses related to OR time (billed in 30-minute increments), BTX-A drug costs (standardized to 100 units), other drugs (excluding BTX-A), anesthesia, preprocedural holding, postanesthesia recovery, laboratory testing, and supplies. Procedural costs in these settings were captured within facility and perioperative accounting categories rather than as a separate CPT-based cystoscopy component. Direct costs were those directly tied to providing services, including labor, medications, and procedural supplies, whereas indirect costs were assigned by the institutional cost accounting system using health system overhead allocation methods and included costs not directly attributable to individual services or supplies, such as building or equipment depreciation, support services, administration, and facility costs.

Mean total, direct, and indirect costs were calculated for each setting and compared using Welch *t* test with significance defined at *P* < .05 with Bonferroni adjustment for multiple hypotheses. To characterize the relative contribution of individual components to costs, average component-level costs were compared within each setting. Variance decomposition was performed to identify drivers of cost variability through both cost dispersion among users and inconsistent use across encounters. Utilization-adjusted variance was estimated as pσ^2^+p(1−p)μ^2^, where p represents the proportion of encounters in which the component was used, σ^2^ represents the variance among encounters using that component, and μ represents the mean component cost among users. The first term captures within-user cost variability, whereas the second captures variability attributable to differential utilization. Relative contributions were calculated by dividing each component’s variance by the sum of component variances within each setting.

## Results

Our final cohort consisted of 322 tertiary OR, 125 HOPD, and 1492 clinic cases. In the tertiary OR, the greatest drivers of total costs were OR time ($1650.81), BTX-A drug‑related costs ($966.71), and postanesthesia recovery ($932.97). Anesthesia ($398.06), supplies ($293.65), non–BTX-A drugs ($217.46), preprocedural holding ($73.24), laboratory testing ($65.57), and electrocardiogram ($27.10) played a more modest role in total tertiary OR costs (Table 1).

**Table 1.** Average Tertiary Operating Room Costs by Item Type

Item type	Sum of direct costs, mean (SD), $	Sum of indirect costs, mean (SD), $	Total costs, mean (SD), $	No.
OR time	1015.5 (475.23)	635.31 (291.83)	1650.81 (711.61)	322
BTX-A (100U)	770.55 (102.47)	196.16 (36.44)	966.71 (134.46)	269
Postanesthesia recovery	669.27 (492.96)	263.7 (214.65)	932.97 (705.18)	322
Anesthesia	253.74 (158.31)	144.32 (79.41)	398.06 (231.16)	322
OR supplies	293.32 (1075.39)	0.33 (4.14)	293.65 (1075.35)	156
Drugs^a^	174.18 (346.19)	43.29 (83.3)	217.46 (429.41)	322
Preoperative	44.91 (8.54)	28.33 (7.83)	73.24 (10.72)	322
Laboratory tests	38.31 (57.98)	27.26 (39.48)	65.57 (96.99)	207
EKG	14.79 (4.61)	12.32 (2.96)	27.1 (7.32)	39
Total	3261.28 (1503.09)	1372.59 (534.51)	4633.87 (1963.09)	322

Mean direct, indirect, and total costs per tertiary operating room (OR) encounter by procedural component reported as mean (SD). Differences between displayed component sums and total costs reflect variable utilization of individual services (eg, medications, urinalysis, laboratory testing, and electrocardiogram [EKG]), which are not performed in all encounters.

a Drugs exclude costs related to onabotulinumtoxinA (BTX-A).

In the HOPD, the greatest drivers of total cost were also OR time ($1772.42), BTX-A drug‑related costs ($712.63), and postanesthesia recovery ($816.82), with anesthesia ($710.95) playing a greater role than in the tertiary OR. Non–BTX-A drugs ($67.29), preprocedural holding ($110.39), laboratory testing ($39.44), and electrocardiogram ($26.98) played minor roles in total HOPD costs (Table 2).

**Table 2.** Average Hospital Outpatient Department Costs by Item Type

Item type	Sum of direct costs, mean (SD), $	Sum of indirect costs, mean (SD), $	Total costs, mean (SD), $	No.
OR time	722.14 (235.18)	1050.28 (294.3)	1772.42 (495.97)	125
BTX-A (100U)	561.73 (33.57)	150.9 (39.45)	712.63 (70.29)	115
Postanesthesia recovery	325.14 (95.58)	491.68 (220.45)	816.82 (309.68)	125
Anesthesia	282.27 (158.84)	428.69 (264.37)	710.95 (414.82)	124
OR supplies	126.96 (187.78)	0 (0)	126.96 (187.78)	125
Drugs^a^	53.02 (31.23)	14.27 (10.26)	67.29 (41)	122
Preoperative	44.87 (8.97)	65.52 (22)	110.39 (29.01)	125
Laboratory tests	22.08 (17.44)	17.35 (12.3)	39.44 (29.57)	82
EKG	14.5 (6.64)	12.47 (3.53)	26.98 (9.76)	17
Total	2707.58 (1457.81)	2355.74 (689.39)	5063.32 (1985.05)	125

Abbreviations: OR, operating room.

Mean direct, indirect, and total costs per hospital outpatient department encounter by procedural component reported as mean (SD). Differences between displayed component sums and total costs reflect variable utilization of individual services (eg, medications, urinalysis, laboratory testing, and electrocardiogram [EKG]), which are not performed in all encounters.

a Drugs exclude costs related to onabotulinumtoxinA (BTX-A).

In the outpatient clinic, the greatest drivers of total cost were BTX-A drug‑related costs ($932.32) and cystoscopic procedure ($733.70) with a minor contribution of urinalysis ($5.73). Of note, item-level costs are not tracked in the clinic and are associated with the bundled CPT 52287 costs (Table 3).

**Table 3.** Average Clinic Costs by Item Type

Item type	Sum of direct costs, mean (SD), $	Sum of indirect costs, mean (SD), $	Total costs, mean (SD), $	No.
BTX-A (100U)	790.86 (87.22)	141.46 (44.33)	932.32 (127.02)	1399
Cystoscopy	629.38 (305.12)	104.32 (38.72)	733.70 (335.34)	1477
Urinalysis	4.84 (1.24)	0.89 (0.14)	5.73 (1.29)	1088
Total	1619.02 (654.28)	279.99 (119.41)	1899.01 (751.13)	1492

Abbreviations: BTX-A, onabotulinumtoxinA.

Mean direct, indirect, and total costs per clinic encounter by procedural component reported as mean (SD). Differences between displayed component sums and total costs reflect variable utilization of individual services (eg, urinalysis), which are not performed in all encounters.

Aside from drivers of direct, indirect, and total costs, we also distinguished how much each component contributes to cost variability across settings. In the tertiary OR, supplies (29.5%), postanesthesia recovery (25.5%), and OR time (25.7%) accounted for over 80% of total cost variability. Alternatively, in the HOPD, OR time (41.2%), anesthesia (29.2%), and postanesthesia recovery (16.1%) played differential contributions to total cost variability. Total cost variability in the clinic was driven by cystoscopy (63.9%) and BTX-A (36.1%) with minimal contribution of urinalysis (0.0%; Figure 1).

**Figure 1.**
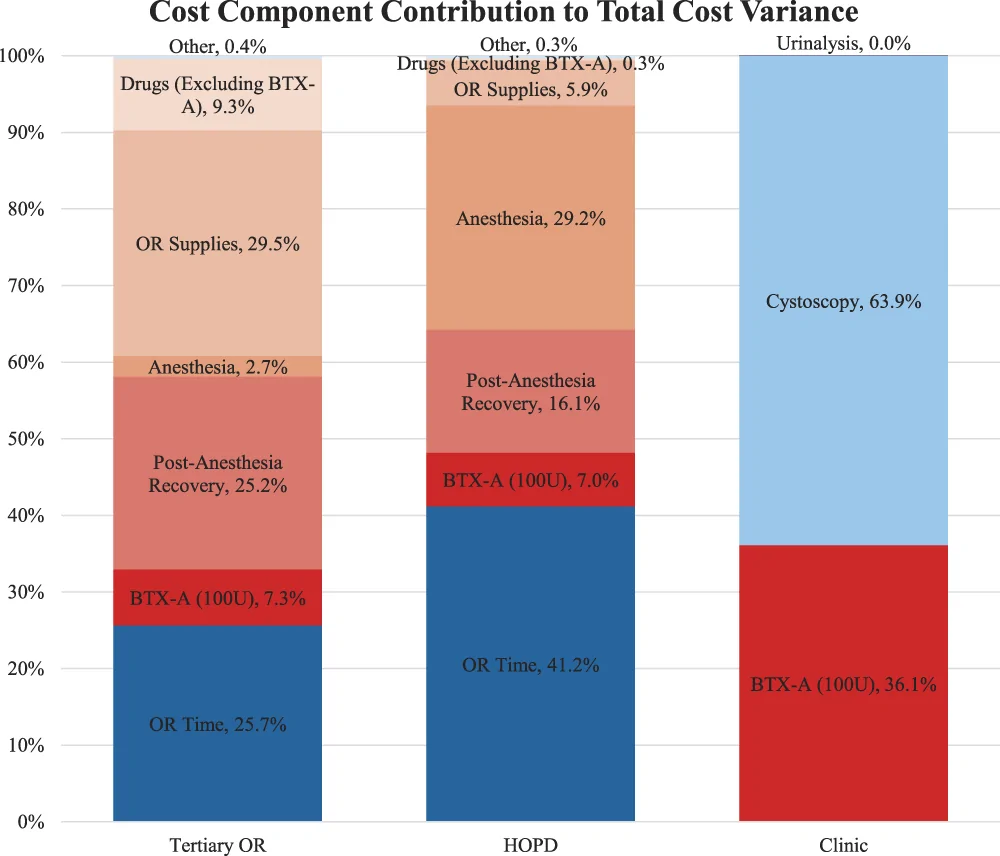
Relative contribution of cost components to total cost variance by setting, accounting for differences in utilization rates. The “other” category includes preoperative, laboratory, and electrocardiogram costs. BTX-A indicates onabotulinumtoxinA; HOPD, hospital outpatient department; OR, operating room.

Direct cost variance drivers in the tertiary OR mainly stemmed from supplies (45.1%) with smaller contributions of OR time (17.5%), BTX-A (7.0%), postanesthesia recovery (18.9%), and non–BTX-A drugs (9.3%). While supplies (23.3%) were also a key variance driver of direct costs in the HOPD, OR time (36.6%), BTX-A (16.1%), and anesthesia (17.0%) demonstrated greater contributions than in the tertiary OR, with minimal variability contribution of non–BTX-A drugs (0.7%). In the clinic, direct costs largely varied because of cystoscopy (68.7%) and BTX-A (31.3%; Figure 2).

**Figure 2.**
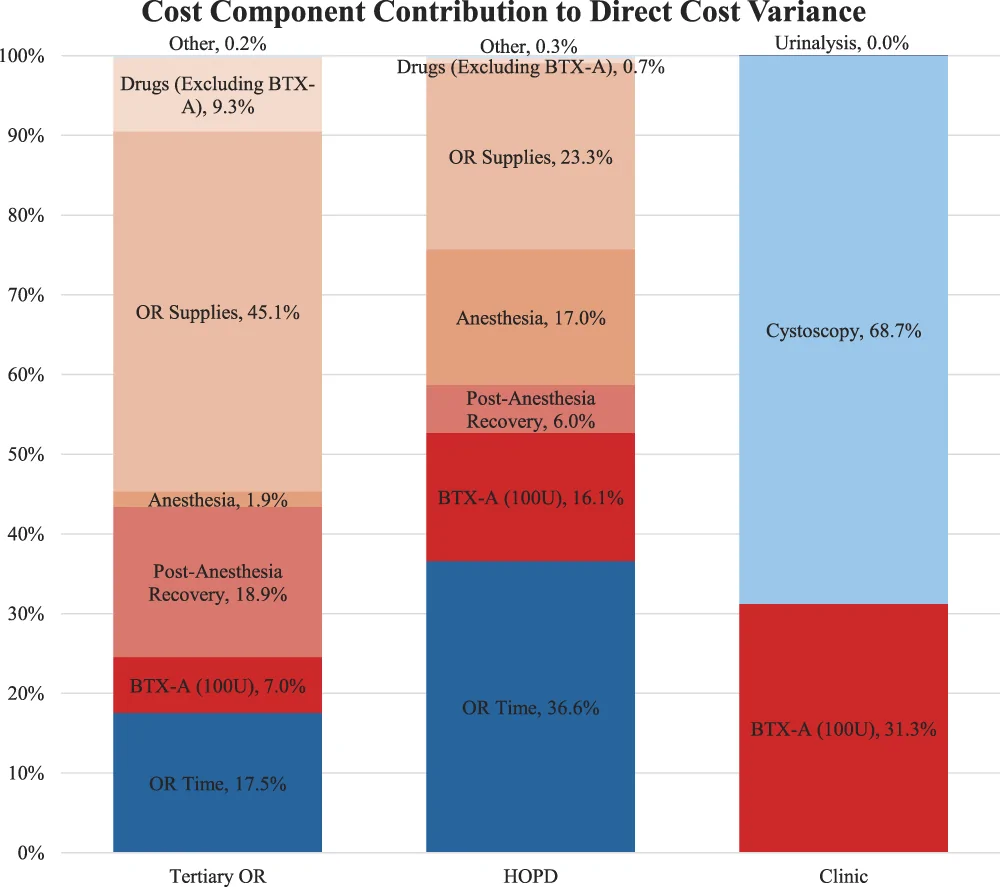
Relative contribution of cost components to direct cost variance by setting, accounting for differences in utilization rates. The “other” category includes preoperative, laboratory, and electrocardiogram costs. BTX-A indicates onabotulinumtoxinA; HOPD, hospital outpatient department; OR, operating room.

Indirect cost variance in the tertiary OR was primarily driven by OR time (56.0%) and postanesthesia recovery (30.3%). In the HOPD, OR time (41.3%) and postanesthesia recovery (23.2%) still drove the brunt of variability, but anesthesia-related costs (33.7%) contributed to indirect costs to a greater degree than in the tertiary OR. In the clinic, BTX-A (65.4%) contributed more to indirect cost variability than cystoscopy (34.6%; Figure 3). While OR-based direct, indirect, and total costs are higher than clinic-based costs, we find that indirect costs do not significantly differ between tertiary OR and HOPD settings (Table 4). Interestingly, tertiary OR direct costs were significantly higher than in the HOPD.

**Figure 3.**
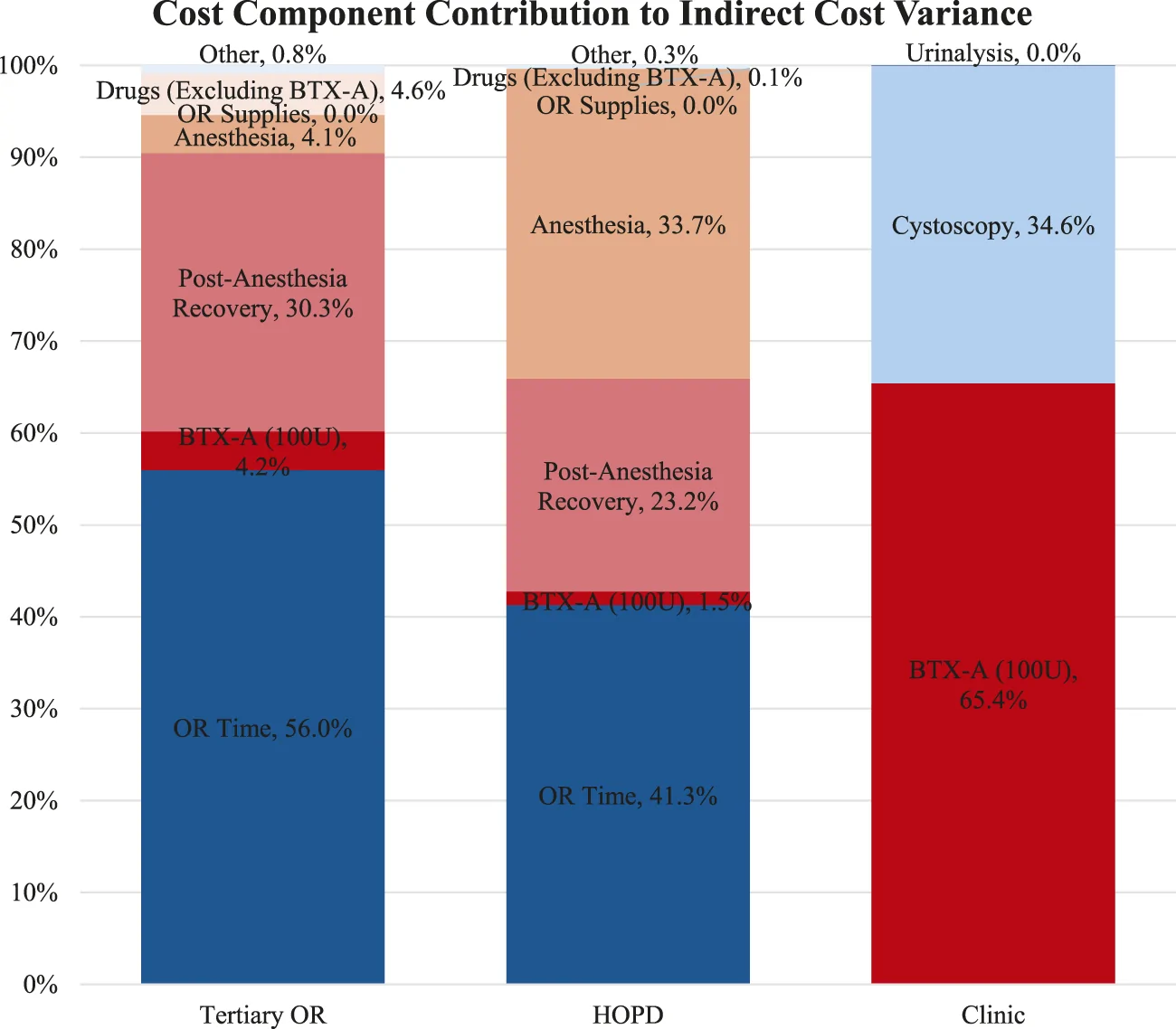
Relative contribution of cost components to indirect cost variance by setting, accounting for differences in utilization rates. The “other” category includes preoperative, laboratory, and electrocardiogram costs. BTX-A indicates onabotulinumtoxinA; HOPD, hospital outpatient department; OR, operating room.

**Table 4.** Pairwise Comparison of Overall Costs by Procedural Setting

	*P* value		
Cost category	Tertiary OR vs HOPD	Tertiary OR vs clinic	HOPD vs clinic
Total costs	**.000429498**	**1.45793E-56**	**1.41503E-13**
Direct costs	**7.55163E-32**	**2.5912E-117**	**3.67498E-64**
Indirect costs	.040631173	**1.29828E-77**	**5.21063E-36**

Abbreviations: HOPD, hospital outpatient department; OR, operating room.

Pairwise comparison of mean total, direct, and indirect overall costs for intradetrusor onabotulinumtoxinA procedures by setting using Welch *t* test with Bonferroni correction of 0.05/9 = 0.0056. Bold values indicate significance.

Given that BTX-A is a costly but necessary component, we examined its cost differences across settings, standardized to 100 units (Table 5). Total, direct, and indirect costs associated with BTX-A acquisition and usage were significantly higher in the tertiary OR than HOPD. While the tertiary OR had higher total and indirect BTX-A costs than the clinic, the clinic had higher direct BTX-A costs. While indirect costs of BTX-A acquisition in the clinic did not differ from the HOPD, the clinic did demonstrate significantly higher total and direct costs than the HOPD.

**Table 5.** Pairwise Comparison of OnabotulinumtoxinA (100U) Costs by Procedural Setting

	*P* value		
Cost category	Tertiary OR vs HOPD	Tertiary OR vs clinic	HOPD vs clinic
Total costs	**4.78867E-78**	**.00012614**	**7.77622E-72**
Direct costs	**4.2097E-100**	**.002500575**	**5.2714E-155**
Indirect costs	**6.46021E-21**	**2.13547E-71**	.015851508

Abbreviations: HOPD, hospital outpatient department; OR, operating room.

Pairwise comparison of mean total, direct, and indirect onabotulinumtoxinA costs per 100 units by setting using Welch *t* test with Bonferroni correction of 0.05/9 = 0.0056. Bold values indicate significance.

## Discussion

Given the exorbitant health care spending in the US, there are ongoing efforts to make specialty care more efficient by reimbursement reform. For example, the Large Urology Group Practice Association developed a bundled payment model for low-risk prostate cancer to incentivize active surveillance rather than more costly treatment options such as surgery or radiation therapy.^18^ The Vanderbilt University Medical Center developed another value-based nephrolithiasis bundled payment program for self-insured employers where providers are not burdened with utilization management.^10^ Initial results from this model revealed improved transparency and predictable costs for employers, decreased financial toxicity for patients, and greater autonomy for physicians.^19^ For such models to be successfully implemented by medical systems at scale, providers need to understand where cost-cutting efforts should be focused to preserve quality. Our large-sample analysis provides, to our knowledge, the first nuanced item-level cost data from the hospital perspective for intradetrusor BTX-A across multiple acuity settings, highlighting supply, operational, and pharmaceutical sources of cost variation.

The most notable findings of this study are the differences in cost variability across tertiary OR, HOPD, and clinic settings, which suggest that cost-containment strategies must be tailored to the driver. Supplies are discrete and directly attributable, making them amenable to supply standardization, vendor contracting, and reduced discretionary use. OR time and recovery costs, in contrast, are workflow dependent and reflected through facility overhead allocation. Reducing these costs may require improving scheduling accuracy, turnover, staffing alignment, recovery protocols, and room utilization rather than item-level cost reduction alone. Anesthesia-related costs represent a separate operational target because they are influenced by sedation approach, staffing structure, and patient selection.

In the tertiary OR, OR time and postanesthesia recovery were major contributors to indirect cost variance, whereas supplies were the dominant contributor to direct cost variance. This suggests that tertiary OR cost containment should combine system-level efficiency such as improving room utilization, minimizing delay, and standardizing recovery criteria with directly traceable interventions such as standardized supply kits and cost-conscious preference cards. Although HOPD direct costs were lower than in the tertiary OR, total costs were higher because of indirect costs, with variability driven by OR time, anesthesia, and recovery. Thus, HOPD cost reduction may depend more on perioperative throughput, anesthesia efficiency, and recovery workflow. This distinction is important because cost for HOPD care is assumed to be uniformly lower than that for hospital-based OR care, yet our findings suggest that the cost advantage of an HOPD depends on how indirect costs are allocated and how efficiently periprocedural resources are used in the context of intradetrusor BTX-A injection.^20^ Clinic-based procedures had a more concentrated cost structure, with mean costs and variability driven primarily by BTX-A and cystoscopy. Cost containment in this setting may therefore focus on drug acquisition, vial waste reduction, payer-specific reimbursement, and administrative burden from prior authorization.^21,22^ Differences in BTX-A cost across settings may reflect institutional variation in drug acquisition, purchasing contracts, inventory management, pharmacy overhead allocation, or payer-specific workflows. Because the cost accounting data set does not identify the specific purchasing pathway for each encounter, we could not determine whether these differences were attributable to manufacturer negotiation, inventory source, or internal cost allocation.

Our unsurprising finding that clinic-based injections are approximately 60% less costly than OR-based injections does not advocate for a site-of-service shift for all patients because cost variability should not be interpreted solely as inefficiency. Patients treated in tertiary OR or HOPD settings may differ from those treated in clinic because of sedation or anesthesia needs, mobility or positioning limitations, anatomic considerations, procedural intolerance, or discomfort. Because these clinical indications were not reliably captured in the cost accounting data set, we could not determine how much variability reflected modifiable practice patterns vs appropriate use of higher resource settings. Providers can also target the direct cost drivers identified in this analysis, BTX-A acquisition, OR supplies, and postanesthesia recovery, through strategic drug purchasing methods such as group purchasing organizations, supply chain standardization, and process mapping by time-driven activity-based costing to identify and eliminate waste.^23,24^ For example, Kapur et al^25^ found that preprocedural phenazopyridine rather than intravesical lidocaine for analgesia yields $98 of savings per procedure, or $24 million annually. Patients can contribute through shared decision-making regarding site-of-service selection and engage in recovery pathways such as discontinuing oral OAB pharmacologic agents after injections. Enhanced shared decision-making support using decision aids that transparently communicate the equivalent efficacy, lower complication rates, and cost advantages of office-based injection has been shown to reduce preference-sensitive procedure costs by approximately 5% and hospital admissions by 12.5% in other clinical contexts.^26^

However, several limitations warrant consideration. Single-institution cost estimates may not generalize to other health systems, particularly given known variation in urologic procedure prices across hospitals, payer classes, and sites of service. Institutions may differ in purchasing contracts, staffing models, facility designation, and overhead allocation methodology; therefore, the relative contribution of cost components may be more generalizable than absolute dollar estimates. Another limitation is the difference in cost accounting between settings. OR encounters included more detailed item-level accounting for perioperative and supply-related services, whereas clinic encounters were primarily reported at the CPT/encounter level. Therefore, specific disposable supply costs in the clinic may not have been separately captured or directly comparable with OR supply costs. Provider-level variation was not evaluated because provider identifiers were not consistently available in the cost accounting data set. Future work incorporating provider-level and patient-level variables could help determine whether cost variability reflects practice patterns or clinical complexity. Payer mix and insurance-specific reimbursement were not included because payer data were not consistently available across all settings, and this study was designed from the provider cost perspective rather than the payer or patient perspective. Future analyses incorporating reimbursement, payer type, and patient out-of-pocket costs would provide a more complete assessment of value across stakeholders. Finally, freestanding ambulatory surgery center (ASC) encounters were not included in this analysis because our institution does not have an ASC. Prior urologic site-of-service work has shown substantially lower Medicare payments in ASCs than HOPDs for outpatient urologic procedures, including urodynamics ($407 vs $1311) and endoscopic bladder surgery ($519 vs $908), suggesting that freestanding ASCs may represent an intermediate-cost setting not captured in our cohort.^20^

## Conclusions

This study demonstrates that cost variability for intradetrusor BTX-A is driven by different components across office-based clinic, tertiary OR, and HOPD settings. Tertiary OR variability was driven by supplies, OR time, and postanesthesia recovery; HOPD variability was driven by OR time, anesthesia, and recovery; and clinic variability was concentrated in cystoscopy and BTX-A. These findings argue that cost-containment strategies should be tailored to the dominant variance drivers within each setting rather than relying on site-of-service substitution alone.
